# Life-Long Aerobic Exercise is a Non-Pharmacological Approach for Inducing Autophagy and Delaying Muscle Atrophy in the Aging Population

**DOI:** 10.14336/AD.2024.0318

**Published:** 2025-03-18

**Authors:** Mingwei Wang, Xiangzhi Wu, Yuyao Jiao, Wenli Yin, Lili Zhang

**Affiliations:** ^1^School of Physical Education, China University of Mining and Technology, Xuzhou, Jiangsu, China.; ^2^School of Physical Education, Jiangsu Normal University, Xuzhou, Jiangsu, China.; ^3^JSNU SPBPU Institute of Engineering Sino-Russian Institute of Jiangsu Normal University, Xuzhou, Jiangsu, China.; ^4^School of Physical Education of Huzhou University, Huzhou, Zhejiang, China.

**Keywords:** aerobic exercise, cellular autophagy, senile myasthenia gravis

## Abstract

Numerous bodily processes deteriorate with age, chief among them being the loss of muscle mass and function. The condition referred to as aging myasthenia gravis impairs older persons' quality of life and is linked to a higher risk of several chronic illnesses. An increasing number of studies conducted in the last several years has demonstrated that moderate exercise can halt this process. Specifically, by promoting autophagy, aerobic exercise helps to postpone the onset of senile myasthenia gravis. In this work, we will explore how aerobic exercise modulates autophagy to prevent muscle aging and examine the most recent findings in this area of study. We discovered that exercise-induced autophagy can effectively balance protein degradation and relieve skeletal muscle atrophy by looking through pertinent literature. Aerobic exercise has a direct impact on autophagy, but it can also delay the onset of senile myasthenia gravis by enhancing blood flow, lowering inflammation, and boosting muscle oxidative capacity. In order to postpone the onset of senile myasthenia gravis, research on the mechanism of action of aerobic exercise in inducing autophagy will be discussed in detail in this study.

## 1. Introduction

Sarcopenia, or senile myasthenia gravis, is a social issue that is becoming more prevalent as the population ages. Sarcopenia is a degenerative condition that occurs with aging that is associated with a progressive loss of skeletal muscle mass, strength, and function. This disease is characterized by a loss of muscle mass, strength, and widespread hypoplasia. Sarcopenia has been identified as a complex age-accelerating geriatric syndrome due to its multifactorial pathogenesis, which includes degeneration of neuromuscular function, disruptions in hormone and protein homeostasis, increased oxidative stress chronic inflammation, and apoptosis, and decreased physical activity [1]. Sarcopenia essentially results from an imbalance that causes net protein breakdown to occur in skeletal muscle as a result of protein synthesis and degradation. Numerous investigations conducted in the last few years have shown that Sarcopenia is characterized by a significant impairment in autophagy-dependent signaling. The intracellular protein degradation system known as autophagy is critical for preserving homeostasis and skeletal muscle mass in vivo. In vivo homeostasis under degrading circumstances and skeletal muscle mass regulation depends heavily on moderate autophagic activity [2].

An efficient intervention to promote autophagy and postpone the onset of sarcopenia is lifelong aerobic exercise. The body's capacity for aerobic endurance metabolism is maintained and enhanced by aerobic exercise, which also raises the mitochondrial content of skeletal muscle, enhances mitochondrial activity, increases mitochondrial aerobic metabolism, and encourages muscle fiber type conversion. Simultaneously, aerobic exercise can preserve the homeostasis of the skeletal muscle's internal milieu by promoting cellular autophagy, eliminating damaged macromolecules and organelles, and supplying substrates for energy production [3]. The pathomechanism of aging myasthenia gravis, exercise and cellular autophagy, and the function and mechanism of aerobic exercise in aging myasthenia gravis will all be reviewed in this paper along with the state of the field's research. It is hoped that this study will guide future investigations and offer theoretical justification for the creation of new intervention approaches and medical procedures.

## 2. Pathogenesis of aging myasthenia gravis

The symptoms of aging-mediated gravis include a notable loss of skeletal muscle mass or strength, a decrease in muscle size, and skeletal muscle fibers. It is described as a complicated multifactorial aging condition including disruptions in hormone and protein balance, neuromuscular function degradation, and neuromuscular dysfunction. A rise in apoptosis and a decrease in physical activity are caused by chronic inflammation and elevated oxidative stress [4]. Dysregulation of several signaling pathways and an imbalance between protein production and degradation are the usual causes of aging myasthenia gravis. The essence can be summed up as follows: skeletal muscle mass is lost as a result of excessive net protein breakdown, which is caused by protein decomposition outweighing protein synthesis [5]. Among them, the autophagy-lysosome system (ALS) and the ubiquitin-proteasome system (UPS) are primarily responsible for controlling protein degradation in skeletal muscle. This is a self-degradation mechanism that uses lysosomal mediation to break down proteins. Long-half-life proteins are the main target of autophagy, a mechanism that selectively breaks down proteins. Intracellular proteins: HSC70 is able to identify and bind to KFERQ signals found at the N-terminus of some cytoplasmic proteins. Proteolytic enzymes break down these proteins after HSC70 facilitates their entry into lysosomes. Extracellular proteins are taken up by endocytosis and then broken down in lysosomes after entering cells. According to recent research, ALS is becoming more and more significant in skeletal muscle as a vital system for degradation that supports cellular metabolism and component conversion. Furthermore, a substantial amount of data indicates that aging-related skeletal muscle abnormalities in autophagy-dependent signaling exist [6]. It is becoming more and more crucial for proper autophagy levels to regulate skeletal muscle mass when degradation conditions are met. Research has demonstrated that UPS overactivation causes an increase in muscle protein breakdown that triggers the onset of myasthenia gravis and is associated with organismal aging. In the meantime, mammalian target of rapamycin (mTOR) stimulates the ubiquitin ligase muscle atrophy F-box protein (atrogin-1) and muscle specific ring finger protein 1 (MuRF1) in UPS and acts downstream of forkhead box protein O (FoxO) to promote enhanced apoptosis and are thought to be the primary controllers of muscle proteolysis [7].

**Figure 1.**
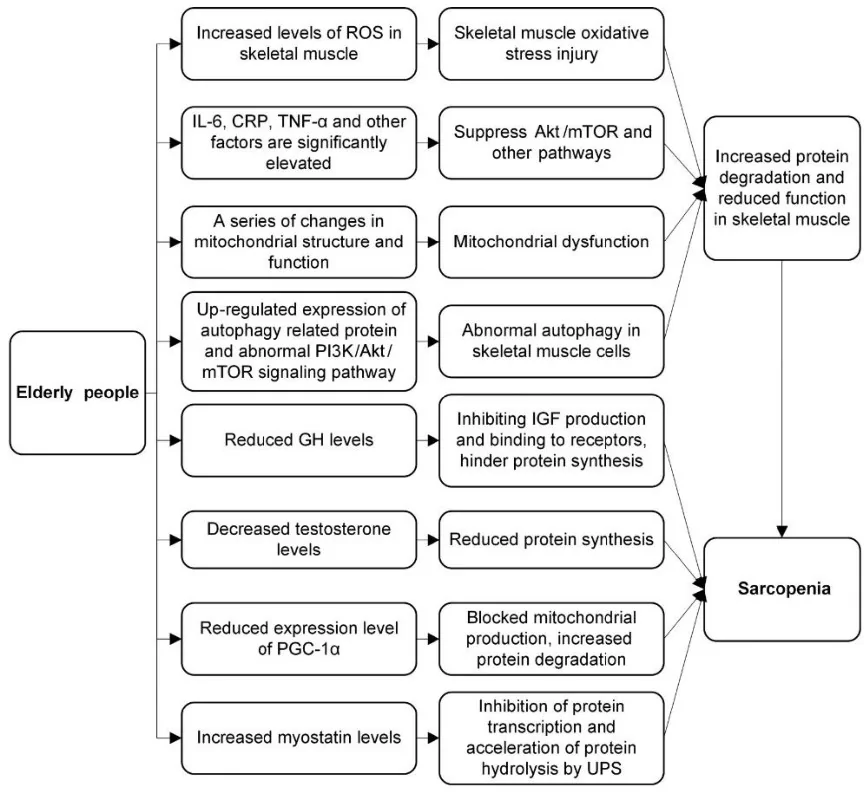
**Schematic diagram of the causative factors of aging myasthenia gravis [8]**. Note: ROS refers to reactive oxygen species molecules produced within living organisms or cells. IL-6: It is a multifunctional inflammatory factor produced by multiple cells that regulates immune response, acute phase response, and hematopoietic function. It plays an important role in the body's antiinfective immunity and autoimmune diseases. CRP: C-reactive protein, is a protein synthesized by the liver. CRP has the functions of regulating immune response, promoting phagocytosis, and activating complement. TNF- α: It is a homologous trimer with a molecular weight of 17 kDa per subunit. It plays an important role in growth regulation, differentiation, inflammatory response, viral replication, tumorigenesis, autoimmune diseases, as well as viral, bacterial, fungal, and parasitic infections. P13K: It is a phosphatidylinositol kinase present in eukaryotic cells P13K plays an important role in cellular signal transduction, participating in various biological processes such as cell growth, differentiation, survival, and metabolism. Akt, also known as protein kinase B, is a serine/threonine protein kinase that plays important roles in cell survival, proliferation, and metabolism. MTOR: is a serine/threonine protein kinase that is an intracellular energy and nutrient receptor that can regulate processes such as protein synthesis, cell growth, and metabolism. GH: Growth hormone. PGC-1 α: It is an important protein that plays a crucial role in sugar and lipid metabolism. IGF: Insulin like growth factor, is a multifunctional cell proliferation regulatory factor that plays an important regulatory role in cell growth, proliferation, and differentiation. UPS: Ubiquitin protease system, which can inhibit protein transcription within cells through various mechanisms.

Aging causes a slowdown in the creation of skeletal muscle cells, an increase in apoptosis and inflammatory regulatory factors, inhibition of protein synthesis, acceleration of degradation, and an increase in apoptosis that leads to myocyte mortality. The inflammatory factors TNF-α (tumor necrosis factorα), IL-1 (interleukin 1), and IL-6 (interleukin 6) are secreted in greater amounts among these factors. These cytokines trigger nuclear factor-κB (NF-κB), which raises UPS activity and hastens the loss of skeletal muscle. They also contribute to the generation of reactive oxygen species (ROS) [8]. Meanwhile, as people age more, oxidative stress rises. It causes a spike in ROS in skeletal muscle, which aggravates protein hydrolysis, activates UPS, and increases downstream production of atrogin-1 and MuRF1 [8]. Additionally, it causes oxidative damage to mitochondrial DNA (mtDNA), which further impairs mitochondrial function and increases the risk of energy deficit, skeletal muscle dysfunction, and senescent myocyte death. Thus, predisposing variables that accelerate the development of aging myasthenia gravis include aging, increased oxidative stress and inflammation, increased apoptosis, negative protein homeostasis, and mitochondrial dysfunction (Fig. 1).

### 2.1 Mitochondrial dysfunction and senile myasthenia gravis

**Figure 2.**
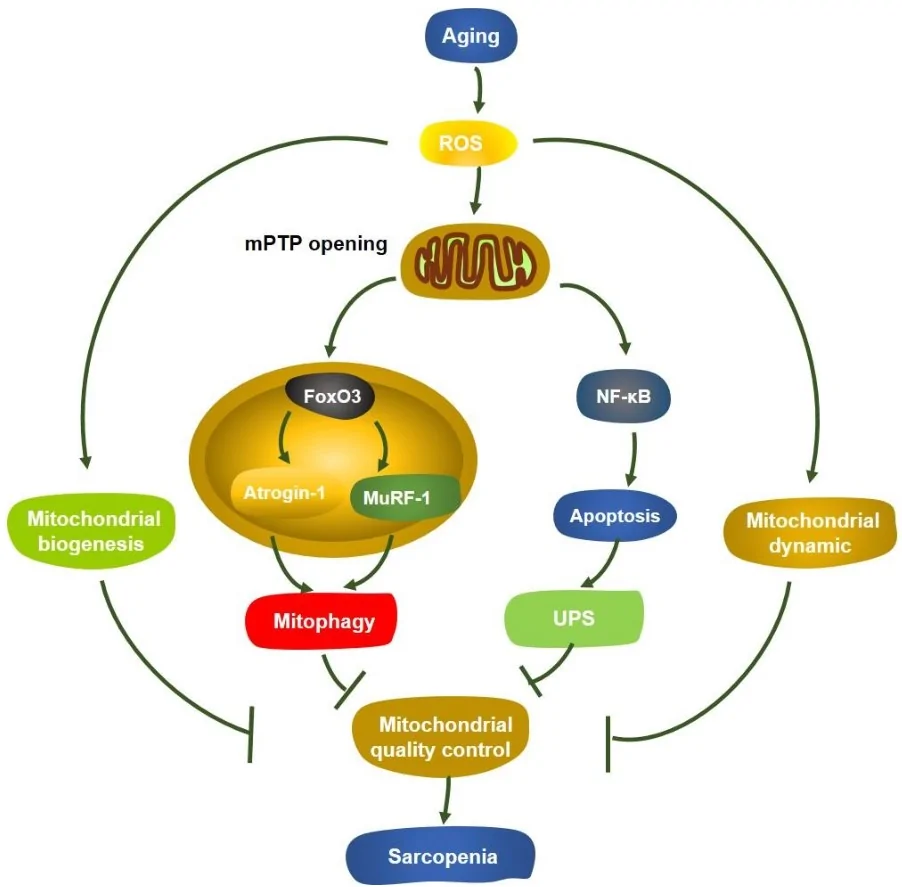
**Schematic diagram of mitochondrial regulation of aging myasthenia gravis [11]**. Note: ROS refers to reactive oxygen species molecules produced within living organisms or cells. Mptp: Mitochondrial permeability transition pore, is a large molecular complex that exists on the inner membrane of mitochondria and is one of the important channels on mitochondria. FoxO3 is a human protein encoded by the FOXO3 gene Atrogin-1, also known as muscle atrophy F-box (MAFbx), is an E3 ubiquitin ligase that mediates protein hydrolysis events that occur during muscle atrophy. MuRF-1: is a 40 kDa protein that can induce ubiquitination of cardiac troponin I NF- κ B: It is a transcription factor that plays a crucial regulatory role in cytokine induced gene expression UPS: Ubiquitin protease system, which can inhibit protein transcription within cells through various mechanisms.

Thorough investigation has revealed a strong correlation between the modulation of mitochondrial mass and the health of skeletal muscle. One of the major pathogenic causes in aging myasthenia gravis has been demonstrated to be mitochondrial dysfunction brought on by age (Fig. 2). Under typical circumstances, homeostasis in mitochondrial mass is maintained by constant dynamic fluctuations in mitochondrial biogenesis, kinetics, and autophagy. Among these, aged myasthenia gravis is significantly influenced by mitochondrial dynamics [9]. Its core apparatus is made up of mitochondrial dynamics-related protein 1 (Drp1) in the mitochondrial division core apparatus and fusion proteins (Mitofusin 2, Mfn2) in the outer mitochondrial membrane. As a result, it efficiently supports the procedures involved in ATP transfer, proliferation, and mitochondrial synthesis and repair [10]. According to studies, skeletal muscle atrophy is directly caused by an imbalance between the processes of mitochondrial fusion and division, which is linked to mitochondrial autophagic breakdown. For instance, compared to young adult skeletal muscle, the expression of fusion and division proteins is comparatively reduced in old skeletal muscle. As a result, mitochondria are crucial for cellular metabolism and, through fusion and division, create a dynamic reticular network in skeletal muscle. By using mitochondrial autophagy to break down and eliminate damaged parts, this network keeps cells in a state of homeostasis [11].

In the meantime, in stress-induced circumstances like senescence, mitochondrial autophagy can be triggered, causing depolarized, damaged mitochondria to be encased in particular phagocytic vesicles and form autophagosomes. As a result, autophagic lysosomes are produced for targeted destruction, which helps to preserve the integrity of the intracellular environment by promptly eliminating damaged mitochondria. It was shown that the primary signaling mechanism of mitochondrial autophagy in skeletal muscle, PINK1 (PTEN induced putative kinase 1)/Parkin, can control the polyubiquitination of damaged mitochondria and a number of useful proteins before autophagic lysosomes degrade them. As we age, the PINK1/Parkin-regulated reduction in ubiquitination will cause a decrease in mitochondrial autophagy activity, which will contribute to the build-up of ROS and damaged materials and affect the mitochondrial activity of skeletal muscle [12].

Redox equilibrium is compromised by aging, which causes skeletal muscle weakness and steady oxidation of cellular constituents, including contractile proteins. Age-related degradation of mitochondrial activity is known to result in increased generation of ROS based on the primary mechanism of organismal aging [8]. Mitochondrial malfunction is a consequence of an imbalance in 11 proteostasis caused by oxidative stress [13]. Senescence is usually accompanied by a decline in mitochondrial kinetic efficiency, with lower turnover, increased apoptosis, and decreased biosynthesis. Eventually, the structural integrity of mitochondria is compromised by imbalances in division and fusion, among other things. Skeletal muscle performance is hampered by the loss of structural integrity in the mitochondrial cell membranes, which also quickens the rise in ROS generation. ROS is a two-edged sword that both causes protein changes and functions as a redox messenger to regulate intracellular signaling. ROS cause lipid peroxidation, protein modification, and direct damage to mtDNA in addition to acting as redox messengers in the regulation of intracellular signaling. In aging skeletal muscle, oxidative stress is also intimately associated with mtROS. Aging reduces the amounts of antioxidant enzymes in skeletal muscle, increases the formation of ROS, and damages mtDNA replication more quickly. This sets off a vicious loop that eventually causes skeletal muscle myopathy by accumulating mtDNA mutations and deletions. It has been shown that as an organism ages, its antioxidant activity declines. Skeletal muscle atrophy is caused by an increase in apoptosis and protein degradation in skeletal muscle cells, which is triggered by ROS-mediated activation of NF-κB and FoxO and downstream expression of atrogin-1 and MuRF1 [14].

### 2.2 Apoptosis and aging myasthenia gravis

One type of planned cell death is called apoptosis. Under normal circumstances, apoptosis can assist in ridding the body of cells that have malfunctioned or are abnormally functioning in order to preserve the dynamic balance of the internal environment and control its relative stability. ROSs, apoptogens, and cytokines can all cause apoptosis. Age-related activation of skeletal muscle apoptosis has been shown to be a factor in both muscle mass loss and malfunctioning of skeletal muscle cells. Both the non-caspase-dependent and caspase-dependent apoptotic pathways are crucial components of the apoptotic pathway. The exogenous apoptotic pathway, which is initiated by signals from outside the cell, and mitochondria-mediated endogenous apoptosis, which is triggered by signals from inside the cell, make up the majority of the Caspase-dependent pathway [15].

TNF-α expression increases in response to an increase in organismal senescence, which in turn activates downstream caspase-8 and caspase-3 activation and initiates apoptosis. On the other hand, the primary source of non-caspase-dependent apoptosis is the reciprocal binding of members of the Bcl-2 family, such as the pro-apoptotic protein Bax and the anti-apoptotic protein Bcl-2. Apoptosis is facilitated by the activation of Bax/Bak aggregates to the mitochondrial membrane [16].

The development of aging myasthenia gravis is largely dependent on the mitochondria-mediated endogenous apoptotic pathway, as recent research has demonstrated. It can be mediated by controlling the amount of AIF that enters myocyte nuclei, causes the outer mitochondrial membrane to permeate and starts the caspase cascade reaction which affects the chromosomes in the nucleus and increases the amount of DNA fragments that break off. Simultaneously, elevated TNF-α-induced apoptosis may trigger UPS, leading to a rise in accelerated muscle protein degradation. This, in turn, encourages proteolysis within skeletal muscle cells and intensifies the loss of skeletal muscle [17]. As a result, as the organism ages gradually, its mitochondrial mass and number diminish and ultimately lead to apoptosis. The apoptotic signaling pathway is activated when skeletal muscle cells produce more ROS. The skeletal cells will therefore ultimately proceed toward apoptosis.

### 2.3 Autophagy and senile myasthenia gravis

Normally, when skeletal muscle contracts mechanically, it releases harmful proteins and senescent, damaged organelles. By removing faulty and malfunctioning organelles and damaged proteins, autophagy acts as a "housekeeper" to preserve cellular homeostasis [18]. Consequently, longevity and optimal health depend on healthy autophagy. But as we age, our tissues change in terms of the lysosomal system's architecture and enzyme activity. Autophagosome activity and quantity decline, and misfolded or missynthesised proteins as well as a high number of senescent organelles build up within the cell and are not eliminated in a timely manner. Thus, the cell's ability to adapt to its surroundings and defend itself is compromised. Numerous aging illnesses are brought on by this. It has been documented that as they age normally, invertebrates and higher species exhibit decreasing autophagy activity. Additionally, it has been discovered that in Drosophila skeletal muscle, the autophagy-lysosomal system's capacity to break down proteins decreases with age. In the aging Drosophila muscle, this causes a gradual build-up of polyubiquitin protein aggregates. Likewise, ubiquitinated protein aggregation and the autophagy receptor molecule p62/SQSTM1 have been noted in skeletal muscle autophagy-specific mutant mice or sarcopenia in myopathy patients [19].

In skeletal muscle of elderly rats, it was observed that the expression of the autophagy-specific protein LC3 was decreased; confirming further that modest amounts of autophagy are maintained during the aging process [20]. It was also discovered in mice lacking the autophagy-specific gene Atg7 in the skeletal muscle. The UPS showed a considerable upregulation of the atrophy-associated proteins atrogin-1 and MuRF1, which had an impact on the myofiber area and muscle mass of skeletal muscle. According to the information above, low cellular autophagic activity is directly linked to the aging skeletal muscle's decreased flexibility and plasticity. On the other hand, increased autophagic activity may worsen skeletal muscle loss by greatly enhancing the activation of skeletal muscle degradation pathways. Moreover, maladaptive stress responses may be activated by autophagy suppression. It could cause extensive protein degradation by boosting the system's activity for protein hydrolysis, impacting the equilibrium of proteins in skeletal muscle. The aforementioned results imply that autophagy deficiencies may be a secondary cause of the aging-related decrease in skeletal muscle mass. One important factor in preventing muscle atrophy is appropriate autophagic activity [21].

**Figure 3.**
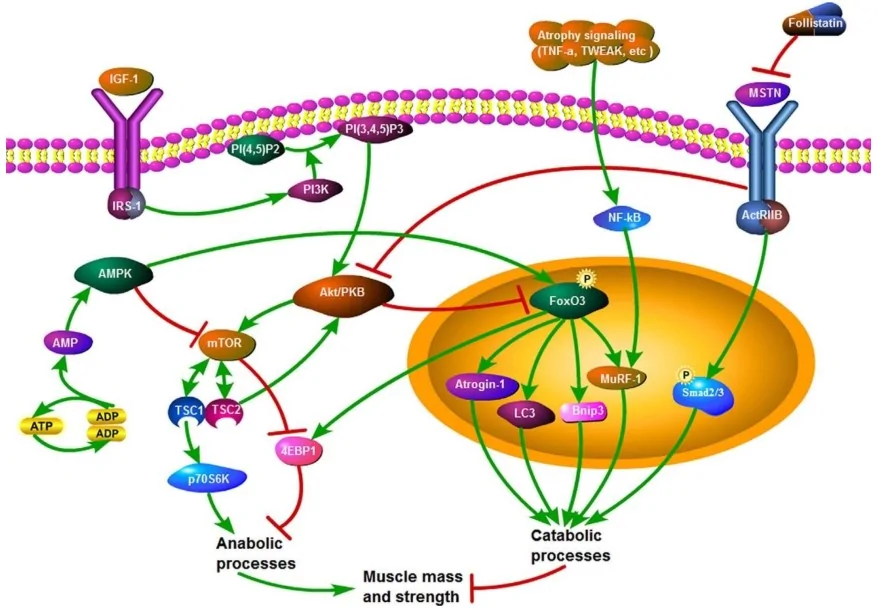
Mechanisms of autophagy regulation of aging myasthenia gravis [7].

According to research, Research has shown that aging myasthenia gravis is a process in which skeletal muscle fibers gradually lose their ability to adapt to changing environments. In short, there is an imbalance in protein metabolism and an accumulation of damaged components as a result of the skeletal muscle protein breakdown rate exceeding the rate of protein synthesis. Furthermore, the activities of UPS and ASL are the primary regulatory variables influencing the process of skeletal muscle protein breakdown [22]. Through UPS, FoxO3a, a significant transcriptional regulator, can raise the myasthenia gravis-associated proteins MuRF1 and atrogin-1. Therefore, controlling the metabolism of proteolysis and impacting the bulk of skeletal muscle. overexpression of MuRF1 and atrogin-1 has been shown to speed up ubiquitination in skeletal muscle, which increases protein breakdown. It decreases the integrity of muscle fibers [23]. Conversely, it has also been discovered in recent years that FoxO3a can stimulate the muscle tissues' autophagy-lysosomal system. During transcription, FoxO3a can enhance the expression of autophagy proteins, including Atg8/LC3, Vps34, Atg6, and Bnip3. These proteins can contribute to protein degradation and preserve the organism's internal environment's homeostasis. Meanwhile, a crucial regulatory protein in muscle atrophy is the transcription factor FoxO3a. For instance, aging may cause an increase in its expression, which raises the activation of the UPS downstream and results in the loss of muscle mass in the skeletal muscle (Fig. 3).

## 3. Exercise and cellular autophagy

### 3.1 Exercise and cellular autophagy regulate whole body health

The body as a whole and the metabolism both benefit from exercise. Frequent exercise can enhance cardiovascular health, muscular mass, mood, and cognitive function in addition to glucose homeostasis. Numerous studies have focused on the adaptive mechanisms of cellular autophagy mediated by exercise. Exercise has been demonstrated to activate the autophagy lysosomal system and the ubiquitin proteasome, two important cellular protein hydrolysis mechanisms, in a variety of human and rodent organs and tissues [24]. All eukaryotic cells have a basal level of cellular autophagy, which is in charge of preserving organelle homeostasis and hence aids in controlling the intracellular environment. As an adaptive response mechanism that is crucial for maintaining cell survival and viability, cellular autophagy can be further upregulated during metabolic stress, which is defined by increased oxidative stress, energy imbalance, or protein misfolding. Conversely, decreased aberrant cellular autophagy levels might result in tissue damage and a lack of cellular stability. Low levels of cellular autophagy have been demonstrated in a number of degenerative illnesses, muscular dystrophy, and even aging. Conversely, in circumstances like persistent muscular atrophy and cachexia brought on by cancer, an overabundance of cellular autophagy could facilitate proteolytic metabolism [25]. Numerous tissues and cell types have demonstrated the induction of cellular autophagy by diverse cellular stresses. These include calcium imbalances, disruptions in intracellular homeostasis, and metabolic stress manifested as oxidation, nutritional shortages, etc. [26]. On the other hand, exercise increases the activation of cellular autophagy by inducing a multitude of stresses. Salminen initially documented an increase in autophagic vesicles' quantity and size in rat skeletal muscle during prolonged, hard exercise in the 1980s.

Furthermore, a plethora of research findings have demonstrated that cellular autophagy is activated in skeletal muscle, and that it is elevated in a variety of human and rodent tissues during exercise. It has been discovered that exercise, on the one hand, causes a rise in oxidative stress, energy imbalance, intracellular calcium, protein misfolding, and cellular autophagy activation. Consequently, angiogenesis, metabolic adaptations, and protein turnover are impacted by cellular autophagy. Exercise performance increased as a result of these adjustments, which also enhanced lipid and glucose balance. The organism's long-term adaptability to exercise is improved [27]. Conversely, poor cellular autophagy results in pathological alterations in mitochondrial structure and function, diminished muscle tone, increased oxidative stress, and loss of muscle strength. The dysfunctional autophagy state of cells could impair sports performance. Research has demonstrated that contractile activity brought on by exercise training stimulates both mitochondrial and cellular autophagy in skeletal muscle. Inadequate cellular autophagy results in decreased exercise performance and compromised metabolic changes brought on by exercise training, including angiogenesis and mitochondrial biogenesis [28].

## 3.2 Mechanisms of action of lifelong aerobic exercise in inducing autophagy

### 3.2.1 Regulation of energy metabolism

Exercise that boosts mitochondrial activity and energy metabolism is called aerobic exercise. As the hub of the cell for energy metabolism, mitochondria can increase the amount of ATP and ROS produced by increasing their metabolic activity. The primary energy source in a cell, ATP powers a variety of metabolic processes. More ATP is produced when mitochondrial activity rises because of the improved metabolic capability of the organelle. ROSs are byproducts of the metabolic activity that occurs within the mitochondria. Superoxide water and hydroxyl radicals are among them. At the right quantities, these reactive oxygen species can function as signaling molecules to initiate the autophagy process. The process of autophagy, which breaks down and recycles intracellular materials, is crucial for preserving the integrity of the intracellular environment. Senile myasthenia gravis is caused by intracellular toxins that accumulate as a result of diminished autophagy activity throughout age. Frequent exercise and physical activity improve blood glucose homeostasis, cardiovascular health, muscle mass, mood, and cognitive function, among other aspects of overall body health and metabolism [29]. Exercise improves nutrient uptake and distribution at the cellular level, creating healthier tissues that are better able to withstand life's challenges [30]. Energy shortages during exercise cause cells to adapt to accommodate increased energy requirements. In order to create new proteins and regenerate organelles, this process requires the degradation of oxidized and damaged proteins in addition to ineffective and malfunctioning mitochondria. For a long time, the molecular analysis of protein synthesis and the replenishment of new organelles has been a top focus for research. Nevertheless, there is mounting evidence that the breakdown of proteins and organelles is just as crucial to cell renewal as production. This shows that during exercise, physiological and biochemical processes are significantly influenced by cellular autophagy.

### 3.2.2 Signaling pathway activation

A number of signaling pathways, including AMPK and mTOR, which are crucial for the induction and control of autophagy, can be activated by aerobic exercise. An essential component of the AMPK signaling cascade, AMPK is a physiological energy sensor. This system is in charge of detecting the energy status of eukaryotic cells and turning them on when there is a shortage of energy. As an intracellular energy receptor, AMPK can respond to low ATP levels and control intracellular energy metabolism to preserve cellular homeostasis. To maintain cellular energy balance, AMPK's primary job is to boost ATP synthesis and block ATP consumption pathways. A key regulator of cell growth and metabolism, MTOR is a serine/threonine protein kinase. In addition to inhibiting catabolic processes like autophagy, it can stimulate the formation of ribosomes and the creation of proteins, nucleotides, fatty acids, and lipids. For instance, when energy is scarce, the AMPK pathway becomes active and inhibits the mTOR pathway, which in turn encourages the induction of autophagy. Another significant factor in the heart's reaction to exercise is cellular autophagy. It was demonstrated that cardiomyocyte autophagy was triggered by running table exercise within 30 minutes. This is connected to the degradation of p62 and the transformation of LC3-I into LC3-II. The Bcl-2-beclin-1 complex must dissociate in order for exercise to activate autophagy in cardiomyocytes. By interacting with Beclin1, bcl-2 can prevent autophagy [31]. There has also been evidence of a rise in autophagy-related proteins (e.g., LC3-II, p62, Bcl-2) in the myocardium in other investigations involving exercise-trained rats [32]. In the meantime, a number of variables, including exercise intensity, duration, and mode, also influence the induction and regulation of autophagy in cardiomyocytes. Therefore, in order to properly utilize the promoting effect of aerobic exercise on cardiomyocyte autophagy and to maintain the healthy state of the heart, an appropriate exercise program needs to be created according to the individual situation and goals.

### 3.2.3 Cellular stress response

In healthy conditions, cellular autophagy is an internal circulatory system that manages and stabilizes the quality of the cell's proteins and organelles. A cellular stress response, including a reaction to oxidative stress and inflammatory processes, can be elicited by aerobic exercise. In order to eliminate damaged cellular components and encourage cellular adaptability and repair, stress responses can trigger autophagic activities. Mice with pathological cardiac hypertrophy and dysfunction are caused by deficiencies in autophagy proteins. The process of protein degradation is accelerated in reaction to cellular stress by autophagy levels, which also releases energy in response to the cell's fluctuating energy and nutritional needs [31]. In disease situations, cellular autophagy is essential for the breakdown of aberrant protein aggregates and organelles damaged by senescence in cardiomyocytes [33].

## 4. Advances in lifelong aerobic exercise to delay aging myasthenia gravis

### 4.1 Sedentary lifestyle and senile muscular dystrophy

Sedentary lifestyles have an impact on skeletal muscle mass, strength, and physical performance as people age. Eight to twelve hours a day are spent inactive by older persons over 60, according to statistics [34]. This way of living dramatically lowers the body's aerobic capacity and speeds up the breakdown of skeletal muscle. It speeds up skeletal muscle loss by causing mitochondrial dysfunction and an increase in oxidative stress. As preventive and treatment, exercise intervention is currently regarded as the most effective form of intervention by both domestic and foreign experts. Individualized and appropriate exercise is a well-established kind of rehabilitation. It can more successfully enhance the organism's health and lengthen life, enhance muscular function and quality of life, and enhance the organism's ability to adapt in older people [35]. Therefore, from a scientific perspective as well as for potential therapeutic applications, it is especially crucial to comprehend the molecular mechanisms underlying senile myasthenia gravis. More effective prevention and treatment outcomes will result from appropriate and successful exercise interventions in the onset and progression of senile myasthenia gravis.

### 4.2 Comparison and selection of resistance and aerobic exercise

Numerous academics, both domestically and internationally, have conducted intervention research on senile myasthenia gravis in recent years. Of these, the exercise intervention primarily compares the impacts of various exercise intensities and types, such as resistance and aerobic training. Additionally, there are some variations in the study's findings. The study found that resistance training is an efficient way to encourage the thickness of muscle fibers. Increases in cross-sectional areas result in an improvement in muscle volume and mass. It improves athletic ability and preserves muscle strength while lowering the risk of falls and fractures brought on by myasthenia gravis [36]. Large muscular groups are used during aerobic exercise, which increases energy production by increasing capillary and mitochondrial density. It boosts muscle endurance and oxygen uptake, enhances muscle responses to exercise, and increases the cross-sectional area of muscle fibers, which slows the loss of skeletal muscle. However, there is usually some risk involved with high intensity resistance training for the senior population. Additionally, senior persons with specific cardiovascular conditions should not use it. A high degree of universal use is also challenging to attain due to the small and restricted selection of resistance equipment made especially for elderly patients. There is no expert supervision for regular resistance training exercises like push-ups, elastic band exercises, and other functional activities. It is challenging to guarantee the necessary load and intensity and to standardize technical motions, which significantly lowers the effectiveness. The efficacy of exercise interventions is significantly diminished by the difficulty in achieving the necessary load and intensity as well as the standardization of technical motions [37]. Because of this, the variety of aerobic exercise is greater than that of strength exercise. It is affordable and resistant to environmental constraints. To stop the onset of aging-related skeletal muscle atrophy, it is more appropriate to promote it to the majority of sick and old patients [38]. At the moment, the majority of research on aerobic exercise intervention for skeletal muscle illnesses associated with aging occurs after the onset of aging. Age-related loss of skeletal muscle mass and strength coincided with the start of osteoporosis in a study using mice that were 18 months of age and given aerobic and resistance exercise. This does not lead to the mice's "burning" of body fat because it restricts the amount and intensity of the exercise that they conduct. Therefore, the impact of weight control is not immediately apparent. In this sense, a healthy lifestyle is a great method to prime the body to begin exercising at a specific load and intensity in early adulthood. It enhances exercise's adaptive capacity, which enhances different tissues' ability to adjust to the effects of aging.

### 4.3 Role and mechanisms of aerobic exercise in senile myasthenia gravis

Exercise has been shown to significantly extend life, according to research. One of its exact modes of action is to enhance cells' ability to repair and defend themselves by enhancing autophagy's functioning state within the body, which has the impact of delaying aging. Mitochondrial kinetic efficiency falls, mitochondrial quantity and quality diminish, and mitochondrial integrity steadily deteriorates with age. Skeletal muscle's aerobic capacity is decreased, for instance, when biogenesis declines, apoptosis rises, and turnover falls. Accordingly, it is believed that apoptotic signaling activation and mitochondrial dysfunction are significant pathogenic contributors in aging myasthenia gravis. Aerobic exercise is a useful intervention because of this. It can maintain and improve the body's aerobic endurance metabolic capacity by increasing the number of mitochondria in skeletal muscle, enhancing mitochondrial activity and mitochondrial aerobic metabolism, and promoting muscle fiber type conversion [39].

Concurrently, there is mounting evidence that the autophagic recycling of cellular constituents is an essential mechanism in the adaptive response of the body to physical activity. Aerobic exercise can efficiently boost autophagic activity to enhance skeletal muscle mitochondrial quality control, govern the mitochondrial energy network, and preserve skeletal muscle metabolic homeostasis while autophagy continues to deteriorate with age. For instance, aging rats in a study experienced improvement following an 8-week aerobic exercise intervention. Their skeletal muscle was discovered to have much higher expression of the autophagy-related genes LC3-II and Beclin1, indicating that cardiovascular training can efficiently counteract the aging-related loss in autophagic activity [40]. Aerobic exercise has been shown to raise AMPK activity in skeletal muscle, block mTOR-mediated phosphorylation of ULK1, and help form the ULK1-Atg13-FIP200 complex, which in turn raises autophagy levels [41]. In addition, aerobic exercise increases one's capacity for aerobic activity by triggering the AMPK/FoxO3a signaling pathway, which breaks down proteins that have been damaged or denatured and produces amino acids and other energy substrates needed for the contraction and metabolism of skeletal muscle. Therefore, it is possible that autophagy's main purpose during aerobic activity is not to build skeletal muscle mass. Instead, it acts as a safety net to keep cells from shutting down and provides a suitable degree of protein regulation to keep the body functioning at an aerobic level. In the meantime, Jiang [42] has previously shown that skeletal muscle cell homeostasis may be preserved and cellular autophagic activity can be efficiently increased in aged rats with aerobic exercise intervention. Nonetheless, varying energy consumption dictates the activation of autophagy; additionally, variations in skeletal muscle glycogen reserve impact the production of distinct autophagic stress. Furthermore, cellular autophagy is induced by exercise, an effective stimulus that triggers a stress response. In order to maintain the homeostasis of the skeletal muscle internal environment and eliminate damaged organelles and macromolecules, skeletal muscle cells are stimulated to increase their autophagic activity. In a similar vein, autophagy is required for long-term aerobic exercise to have its cumulative health benefits. Furthermore, autophagy in skeletal muscle cells has distinct adaptive responses in response to prolonged, consistent aerobic exercise; these responses are primarily basal autophagy rather than stress responses.

## 5. Conclusions

This study examined how exercise regulates aging, with a focus on how lifetime aerobic activity induces autophagy, which delays the onset of senile myasthenia gravis. Lifelong aerobic exercise can successfully postpone the development of symptoms associated with myasthenia gravis and the loss of skeletal muscle mass by boosting autophagic activity. Nevertheless, more thorough research is still required to understand the exact mechanism by which lifelong aerobic exercise induces autophagy. Further research can concentrate on investigating additional signaling pathways and molecular mechanisms associated with autophagy in order to offer theoretical backing for the creation of more potent therapeutic strategies to postpone the aging process of myasthenia gravis.
